# Levers and obstacles for implementing public engagement practices in electricity grid development

**DOI:** 10.1016/j.heliyon.2024.e34955

**Published:** 2024-07-23

**Authors:** Evan Boyle, Alexandra Revez, Aoife Deane, Brian Ó Gallachóir

**Affiliations:** a MaREI Centre, Environmental Research Institute, University College Cork, Ireland; b School of Engineering, University College Cork, Cork, Ireland

**Keywords:** Public engagement, Energy transitions, Infrastructure development, Citizen participation, Reflective practice, Grid development

## Abstract

The scale of change required through the development of new energy infrastructure throughout Europe is vast. The societal dimensions of the energy transition are increasingly recognised as centrally important and approaches to infrastructure development which seek to incorporate such considerations are warranted. EirGrid - Ireland's national electricity transmission operator - through their own historical context, have undergone a journey to develop new strategies for citizen and community engagement with relation to energy grid developments.

Here, we reflect upon this journey, situating it within their previous failures and the national context. This process of reflective practice seeks to provide findings for other organisations internationally undertaking a journey towards establishing new engagement practices. The establishment of such practices is critical for enabling deeper societal engagement on the energy transition. A research gap exists in relation to the organisational development of new public engagement practices within institutions tasked with developing infrastructure associated with the energy transition. This creates a challenge whereby ever-increasing calls for public engagement are made, but no lessons exist with relation to how such new practices can be embedded within an organisational strategy. We contribute to this space through answering the research question: what are the key levers and barriers for organisation change towards new forms of public engagement in infrastructure delivery?

The reflections outlined through this paper have been provided by individuals in different positions across the organisation. The paper develops key findings which add to the literature in relation to levers and obstacles for implementing public engagement and associated factors.

## Highlights

• Barrier and drivers for the development of public engagement strategies for infrastructure delivery.
• Reflective practice process across a TSO to provide an analysis of organisational change in relation to public engagement.
• Highlights the tension between speeding up the low-carbon transition and engaging citizens in transition processes.
• Public engagement must be strongly embedded within organisations to influence the technical aspects of project delivery.

## 1 Introduction

Plans are in place across Europe to decarbonize energy systems with increased importance placed on the generation of renewable electricity. Due to this, considerable grid modernization is needed in the years ahead. Over the past decade, the European Union has seen a 12 % increase in its transmission grid. Despite these developments and coupled with the need for increased capacity associated with electrification, grids are ageing which poses reliability risks and safety concerns. A call has emerged for the active fostering of societal support for grid development through active public engagement whereby best practices can inform strategy within grid developing organisations [1]. At a broader level, public engagement has been recognised as essential for achieving societal transformation associated with climate action [2]. The importance of the societal dimensions of developing energy infrastructure is increasingly acknowledged, and as such experimentation with societal engagement in grid developments is currently taking place, although tentatively [3]. In this paper, we are examining the reflections of practitioners paving the way for organisational change and implementing new forms of public engagement in infrastructure delivery. Our primary focus is to identify the key drivers and barriers for organisational change in this context. Moving from policy to implementation is a key development for the integration of deliberative processes into governance. We reflect upon EirGrid, as the national grid operator, on its journey to developing new and innovative engagement strategies for grid development in the Irish context.

A recent IEA report [1] on public engagement in energy infrastructure found that no studies to date have investigated the drivers and barriers of institutions engaging with citizens and communities. Beyond this, no research has taken place which highlights the organisational development of new public engagement practices within institutions tasked with developing infrastructure associated with the energy transition. This creates a challenge whereby ever-increasing calls for public engagement are made, but no lessons exist with relation to how such new practices can be embedded within an organisational strategy. In providing reflections from within a TSO, we develop knowledge in this space. While best practice is often called for, the context specific nature of engaging with diverse publics gives the need for a more measured approach whereby “good practice” can inform the ways in which citizens and other stakeholders are engaged with [4]. Previous work has sought to outline good practice for community engagement within public bodies and state agencies. Within this, some common practice guidelines emerge including transparency, wide and early engagement, building local profiles, and pulse checks. Alongside this, however, is the need for flexibility, in responding to different contexts, circumstances, and changes throughout a project timeline. This is often at odds with the time sensitive nature of established grid development strategies [4]. While this time consideration exists, there is a recognition within the literature of the need for stronger participatory mechanisms is warranted due to citizen opposition to developments [5]

The call for greater participation is premised on the considerable and complex political challenges faced at a global scale, such as polarization, disinformation, geo-political tensions, and climate change [6]. Theoretically, participation can be seen from two main perspectives; normative participation whereby the focus is on the process and the democratic right of citizens to participate, and pragmatic participation or participation to an end, to achieve a better quality of decision making [7]. Normative participation is concerned with the rights of citizens which can lead to collective action, social inclusion, empowerment, transparency, and accountability. Pragmatic participation is concerned with participation as a mechanism to improve decision making and prevent strong opposition. As noted, “participatory governance is expected to contribute to improving the ‘quality’ of decisions by incorporating locally held knowledge” ([8], 198). This expectation may, however, be unfounded and participation may not necessarily lead to a better quality of outcome. The quality of the engagement process is centrally important to the quality of the outcome, and recent research has suggested that a prioritisation of process and relationship building over a binary outcome orientated logic of engagement is needed [9].

Within the literature, there has been a move towards interpreting socio-technical transitions through the lens of institutionalism, i.e., the study of institutions and their impact on aspects of society [10]. Some key questions for consideration when looking at socio-technical transitions nationally through the lens of institutionalism are relevant such as; the nature of embedded institutions and willingness to change behaviour, how the institutional environment shapes policy discourse, making, and implementation in the energy sector; and how does the institutional environment shapes the distribution of agency among different stakeholders and the willingness to experiment in this regard [11].There has been a recent development within Irish policy towards citizen engagement on energy transitions and climate action more broadly [12]; [13].

The Climate Action Plan [13] explicitly referenced the importance of citizen engagement, and through the National Dialogue on Climate Action sought to give space for different aspects of Irish society to have their voice heard in Ireland's decarbonisation and sustainability transitions. The need for systematic and active engagement with different actors within Irish society, at both local and national levels, was recognised within the Climate Action Plan as centrally important, with the National Dialogue on Climate Action referenced as the central mechanism through which this would be achieved. On from engagement at the national policy level, research has emerged which seeks to investigate the role of engagement processes in the development of climate related infrastructure to implement decarbonisation strategies (Boyle et al., forthcoming).

This paper reflects upon the implementation of new engagement strategies for electricity grid development through an analysis of EirGrid and their emergent practice, answering our central research question: what are the key levers and barriers for organisation change towards new forms of public engagement in infrastructure delivery? The structure of the paper is as follows: firstly, we outline the contextual specifics of EirGrid, as Irelands transmission system operator, in relation to public engagement. We briefly outline theoretical insights in relation to both engagement and organisational change which support the development of this paper. Following this, the methods section outlines the use of a reflective practice process. On from this, we develop results related to the reflective process in relation to the engagement journey so far, levers and obstacles to public engagement, and conclusions. In the discussion section, we focus on the need for unified approaches to public engagement at a cross-organisational level to deliver on the energy transition, the tension between meeting climate targets and engaging with citizens and implementing new engagement processes at an early stage. Finally, we draw out conclusions and implications for future developments in relation to sustainability and infrastructural delivery.

## 2 Context: Developing a strategy for public engagement within a transmission system operator

EirGrid's journey is deeply connected to the Irish socio-political, economic, and even geographical position as an Island country in the periphery of Europe. Yet, the story of overreliance on technical and engineering solutions to deliver energy grid upgrades and public protest emerging from overreliance on such methods is not a specific Irish issue and we see this problem replicated across various other countries [[14], [15], [16]].

Decide-Announce-Defend models or eco-authoritarianism style delivery of grid projects have led to a lack of agreements over deployment and to costly delays in the development and cancellation of projects [15]. Contrary to more reductive views of communities as too focused on local issues and incapable of understanding the ‘big picture’ [17] we see from this study how it is possible to include some innovations that provide a more integrated approach to manage the delivery of grid expansion from a multiple scale and stakeholder perspective. The emphasis of these reflections as coming from an Irish practitioner's journey to deliver timely energy system change can be interpreted as an invitation for others to share, reflect, or critique this geographically bound study and its applicability elsewhere.

Since 2006, EirGrid has acted as the transmission system operator for the electricity system in Ireland. Operating to manage the flow of power across the grid, and to plan for its future development, EirGrid is a state-owned company, licensed by the Commission for Regulation of Utilities in Ireland (CRU). Following the establishment of the Single Electricity Market (SEM) in 2007, EirGrid together with SONI in Northern Ireland took over operation of the wholesale electricity market in Ireland. Both EirGrid and SONI are owned by the parent company EirGrid Group. While the electricity transmission system is operated by EirGrid, it is owned by ESB. The distribution system is owned by ESB and operated by ESB Networks. ESB Networks is responsible for the maintenance, repairs, and construction of the grid, while EirGrid is responsible for the future planning and development of the grid [18].

Mullally and Byrne [19], using an analysis of mediated narratives, outline some of the historical considerations in relation to EirGrid, grid development, social acceptance, and community engagement. *Grid25 - A Strategy for the Development of Ireland's Electricity Grid for a Sustainable and Competitive Future* was established by EirGrid in 2008 as an investment program to update and modernise the transmission network. The centrality of infrastructure upgrades was highlighted again in 2010 through the publication of the Government Policy Statement on the Strategic Importance of Transmission and Other Energy Infrastructure. The importance of social acceptance, as outlined within the energy transitions literature [20], was beginning to emerge in Irish policy following this as outlined by a research report published by the National Economic and Social Council [21] which linked best practice on social engagement internationally and in Ireland.

EirGrid, mindful of the importance of public acceptability, developed a public consultation document—Project Development and Consultation Roadmap [22] which was heralded as a significant development for community consultation in Ireland [23]. Despite this, however, from 2013 onwards, EirGrid became embroiled in controversies related to several projects seeking to construct 400-kV overhead lines and pylons across several regions in Ireland. “Grid Link”, from Munster to Leinster; “Grid West”, across Connaught, and the “North-South Interconnection”, between the Republic of Ireland and Northern Ireland all saw the mobilisation of significant community-based opposition and the networking of different oppositional groups within this. 35,000 submissions were made related to grid infrastructure in January 2014 as a part of what came to be one of the largest community mobilisations since the 1970's [19].

The approach to opposition centred on a call for the undergrounding of pylons, a potentially more economically expensive approach, with costs often incurred by the end user. Despite this, undergrounding often increases public acceptance due in part to aesthetic favourability, with people often unaware of the impact of undergrounding on landscape and health [24]. Throughout the oppositional campaigns related to EirGrid's proposed updates, media coverage was heavily focused on the sense that communities were not being listened to and any notion of engagement was tokenistic [19]. This, however, is not beyond the pale, with a recent study finding tokenism to be a common form of participation within energy transitions for stakeholders and lay people [25].

The controversy around the updating of the electricity transmission network and mobilised opposition to the development of pylons led, in 2014, to the establishment of an independent panel to fully review the undergrounding of cables. Alongside this development, “EirGrid announced details of compensation packages for landowners and funds for affected communities. However, the Pylon Alternative Alliance characterised the compensation package as premature” ([19], 10). Distrust in institutional bodies, their motives, and the information they share are central components of the trust gap, which is often evident in energy transitions [26]. By March of 2014, 70 % of people were in favour of underground alternatives to put a stop to the erection of pylons across the countryside [27].

Following this widespread mobilisation, EirGrid reviewed its approach to public consultation, launching a working paper *Your Grid, Your Views, Your Tomorrow* on grid development strategy in March 2015. The use of advanced smart grid technology through “series compensation” whereby additional power can be transmitted through the established grid, assisted in preventing the pursuit of new developments as initially outlined in Grid25. This paper outlined the TSO's shift in strategy towards doing “more with existing grid and make it work harder - before we build new transmission infrastructure ([28], 5).

In 2017, following on from the 2015 working paper, EirGrid revised its grid development strategy committing to the use of new and emerging technologies to enhance the capacity of existing grid infrastructure. Despite the importance of innovation, the increased use of offshore wind and ocean energy moving forward will require not only grid updates but new grid developments. The importance of community engagement within the organisation at this time was noted; “in recent years, EirGrid has developed its capacity for community engagement, including by opening a number of offices and information centres in regions in which new grid infrastructure is planned” ([18], 10). EirGrid also published a document to encourage new forms of public participation in grid development moving forward.

**Fig. 1:**
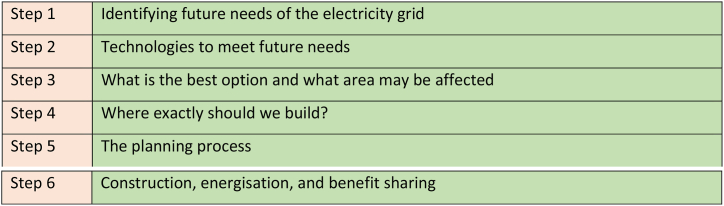
Six-step project development approach for electricity grid development.

At a theoretical level, while no single definition of public engagement can suitably encapsulate the range of forms which it takes, it is being more deeply developed in relation to emergent conceptions of citizenship over passive notions of consumer choice due to the increasing relevance of societal dimensions of transition processes and the political and social factors contained within [29]. Defining public engagement as a fluid category and as a continuously shaped and enacted concept across various scales and places can significantly broaden the scope for engaging various emergent ‘publics’ in energy system change [29]. Equally, the systemic turn in deliberative democratic theory recognises that citizen participation involves diverse actors and occurs in multiple locations, such as civil society gatherings, social movements and the media [30]. This focus on engagement over various fora and across institutional boundaries calls attention to more diffuse, dynamic, and webbed engagement constructs that cannot easily be reduced to solitary and situational events or people [30]. This document announced the organisations new approach to consultation whereby the range of different stakeholders they would engage with were referenced. This included the public (members of local communities, local authorities, and elected representatives), other organisations (businesses, government departments, representative organisations), and landowners (individuals and companies who own land). The document invoked the company's commitment to the Aarhus Convention with regards to public participation in environmental decision making. A six-step approach to project development was outlined within this document as represented below. Public engagement is open through Step 1–4 before going to the planning board in Step 5. At the final step, EirGrid no longer seeks public views on the project at it goes under construction (see Fig. 1).

In 2020, EirGrid set about establishing a new strategy for public engagement. An internal team was established that outlined goals and pathways to standards before undertaking a comprehensive review of the topic of public engagement informed by independent reports conducted through external consultants. The updated strategy for effective public engagement focused on social acceptance - “work towards solutions that have landowner and public support”; capacity - “increase our public engagement capacity and invest in our people and tools; and partnerships “renew and revitalise our existing alliances - and develop new ones” ([31], 9).

EirGrid published “Shaping Our Electricity Future” in 2021, providing a roadmap for achieving renewable ambition in Ireland. The document was prepared using a mixed-method consultation process in both the Republic of Ireland and Northern Ireland over a 14-week period. Over 100 events were held, and 500 submissions were received as part of the process to inform the network development approach to be undertaken by EirGrid in setting out to reach 2030 targets. The public consultation feedback outlined the importance of regional and rural community benefit, community ownership options, microgeneration, and social acceptance, amongst other factors [32]. This step towards deliberative dialogue between EirGrid and public stakeholders in strategy development is representative of the wider governance trend. This paper reflects upon EirGrid's organisational journey towards developing new public engagement strategies, procedures, and processes.

Lewin's foundational change management model, which outlines change across three distinct phases, involves the disentanglement of previous practice, the change taking place, and embedding new practices through a process of permanentization [33]. Beyond this linear pathway, more recent complexity theories add furtherdepth, whereby processes of change are often emergent with small changes having the potential to lead to larger system level outcomes [34]. These two fields of theoretical analysis related to organisational change offer opposing points on a spectrum representing linear and non-linear change. The work of John Kotter [35], developed through his 8-step change model (see Table 1), is referenced through the different stages of the process as reflected upon by participants. Within this the importance of unintended outcomes as represented by complexity theories is also noted.

**Table 1:** Adapted from Kotter [35] 8-step Process for leading change.

Creating the climate for change	Step 1: Create Urgency
	Step 2: Form a powerful coalition
	Step 3: Create a vision for change
Engaging and enabling the organisation	Step 4: Communicate the vision
	Step 5: Empower action
	Step 6: Create quick wins
Implementing and sustaining change	Step 7: Build on the change
	Step 8: Make it stick

## 3 Methods

A process of reflective practice has been developed with members of EirGrid and researchers from MaREI to review the organisational journey towards developing new public engagement strategies, procedures, and processes in relation to the development of electricity grid infrastructure. This has been done to highlight learnings from individual experiences that can be shared at a cross-institutional level to inform similar journeys within other organisations. Alongside this, it can act as a process through which internal learning can take place. Reflective practice has been championed for its transformational potential in relation to professional development [36].

There are two distinct theoretical approaches from which reflective practice can be developed. Firstly, the foundational rationalist-technicist, which applies a systematic approach to attaining knowledge using rational analysis and empirical observation; and secondly, the experiential-intuitive model which captures special expertise and intuitive processes, missing from the rationalist-technicist model [37]. Clarity concerning the approach taken is needed to guide the process. When discussing reflection, we must be clear about what we understand the process to be [38]. Theoretically, this work has been grounded in the experiential-intuitive model of Schön [39,40]. Despite the usefulness of applying Dewey's model [41], this work has used the experiential-intuitive model to outline the special expertise of individuals within EirGrid and the journey towards deliberative processes which the organisation has been undertaking since 2013. The individuals who are taking part in reflections within this paper are made up of a cross-section of the company, to represent the engagement journey at different scales within the organisational structure.

Critical reflection is a process which involves contemplating one's own practice and critically analysing the development of different processes and skills to develop new approaches moving forward ([42], 831). EirGrid's journey towards deliberative processes, and personal experiences of it, provide a fertile foundation from which wider learnings can be gained about the need for further public involvement in energy transition processes. The constant comparative method of analysis [43] was deployed across the seven reflections developed by participants. Selective coding was used to understand the themes with most pertinence to the development of public engagement strategy within the organisation. This created two main groupings: internal levers and barriers and external levers and barriers. Alongside these, more general reflections aligning with a chronological depiction of the process within the organisation were developed (see: Fig. 3).

At a broad philosophical level Soren Kierkegaard ([44] ) saw reflection as a fundamental aspect of the human condition, with no serious action possible without it. Yet, the Danish philosopher saw the dangers of his present age and reflection, which goes on indefinitely, towards an age of inaction. There are dangers when undertaking a process of reflection that it becomes disconnected from reality. Reflection then, “seems only to be of value if it results in something else, in … resolution” ([45], 14). In seeking to reflect upon EirGrid's journey towards more deliberative processes, guiding questions have been designed with this sense of resolution in mind. How can this process of reflection inform moving forward rather than just document looking backwards? The concept of reflection-for-action “refers to the process of planning, thinking ahead about what is to come, so that we can draw on our experience (and the professional knowledge base implicit within it) in order to make the best use of the time resources available to us” ([46], 317). Here, we can move beyond ontological critiques of reflection to guide a process towards institutional capacity building.

**Fig. 2:**
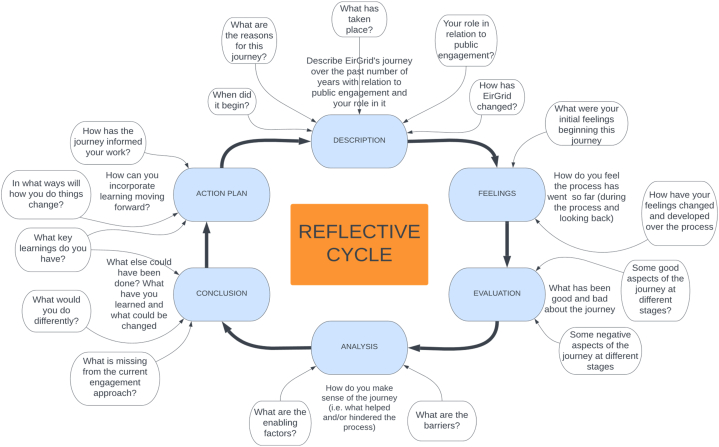
Graphical outline of Gibbs [51] model of reflection, adapted with descriptors for EirGrid case.

The process of reflection has been informed by Gibbs’ six-stage model of reflection. This model is a well-established approach to reflective practice within the literature [47]. The approach has garnered some criticisms for failing to facilitate high level critical reflection [48,49], however the simplistic step-by-step process it provides can assist professionals in undertaking reflective practice for the first time. Questions have been raised about the timeframe over which a reflective practice intervention occurs, with suggestions that long-term reflective interventions may prove more valuable for learning compared to shorter interventions [50]. Here, we have designed a six-step reflective processes (Fig. 2) informed by Gibbs which takes place over a two-month timeframe with each respondent journaling thoughts over each category. The Gibbs model has been built upon to include specific questions (see Fig. 2) in order guide the reflections beyond the broad foundations outlined by the model. Building on this, the incorporation of specific questions (Fig. 2) is used to support the respondents due to the novel nature of such a reflective endeavour within the organisation.

**Fig. 3:**
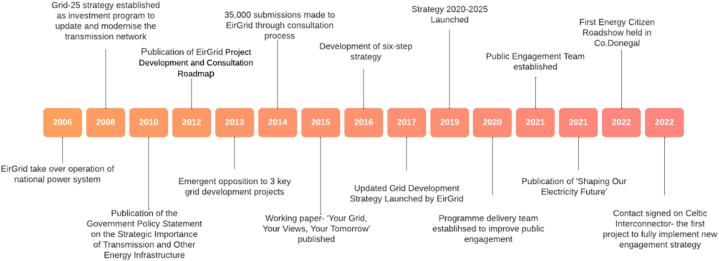
Key milestones in EirGrid Public Engagement Journey 2006–2022.

The reflective practice process was completed by seven individuals working across different levels of the organisation. These were a community liaison officer, chairperson, non-executive director, senior planning officer, head of public engagement, external affairs, and head of network development. Experience within the organisation ranged from 2 years to 15+ years. The gender balance represented was 4 male 3 female participants.

## 4 Results

While a call has emerged for best practices that can inform strategy within grid developing organisations towards active fostering of societal support for grid development [1], here the focus is given to outlining one individual organisation's journey related to the redevelopment of a public engagement strategy. This may inform the creation of best-practice guidelines through comparative analysis. Within the results presented here, the reflections provided by different individuals within the organisation are outlined across three themes: the journey so far, the levers and associated barriers, and conclusions with recommendations and next steps. The reflections were anonymised and coded numerically 1–7 for the different individuals. This process took place between 2022 and 2023. Some biases or conflicts of interest may exist regarding undertaking such a reflective practice within an organisation with active staff members. To give an open space for critical reflection, the reflections provided are not directly attributed to individuals as this would make them clearly identifiable within the Irish context (e.g. chairperson etc.). This was outlined within the ethics form provided to participants. The findings from practitioner reflections may be limited as they heavily depend on their willingness and ability to evaluate their practices. While the practitioners we have worked with have been open and cooperative during the research process, there may still be limitations or biases in how they assess their performance. Despite these limitations, we believe that the results can be useful in other contexts. Many European countries, including Ireland, have faced challenges in fully embracing more extensive forms of public engagement, and some are just beginning to explore this area. Therefore, there is value in learning from the experiences of EirGrid, as shared by the practitioners involved, as it can provide valuable insights and opportunities for others.

The factors and processes outlined are situated in reference to Kotter's [35] 8-step theory of organisational change. The reflections provided in 4.1 were outlined in the “description” and “feeling” phases of the reflective cycle (see Fig. 2). For section 4.2 the “evaluation” and “analysis” phases. The “conclusion” and “action plan” are not directly represented in results, but informed the two sections below and are used in shaping the discussion in section 5.

### 4.1 Reflections on the engagement journey so far

EirGrid's early experience as an organisation was marred by negative public responses concerning project development associated with Grid 25. While criticisms can be directed towards the way consultation was approached, this must also be situated within the wider context. By 2013, the nation had been living with the reality of austerity for four years. As Ó Riain [52] illustrates, the most prominent feature of Ireland's economic crash was “the steady erosion of public trust and political legitimacy, even in the absence of widespread disruptive protest” (2014, 259). EirGrid were part of this, creating the urgency needed for change:

*the Grid 25 project, particularly the Grid Link project, had driven a huge amount of animosity towards the company. Communities were looking for causes to back to kind [of] give establishment Ireland a poke in the eye.* (5)

*the volume of national scale opposition to this was huge because people didn't understand why it was happening … we did have to look deep into ourselves because nothing was getting built. The [EirGrid] name was toxic* (6)

While widespread disruptive protest - as was seen in other European contexts - was absent, locally bound mobilisations, such as those against proposed pylon developments were present. The Right 2 Water campaign (2014-16), in opposition to domestic water charges, followed a similar logic and has been presented as a clear illustration of some of the citizenry's discontent towards austerity measures and the political class [53]. Despite the political and economic landscape in which the opposition to EirGrid projects emerged, there was a clear need for a fresh approach from an internal perspective within the organisation. In advocating for a more interconnected and interactive definition of public engagement, recent research has highlighted the necessity for a new dialogue. This dialogue should be less concerned with ranking the merits of specific public engagement approaches and more focused on understanding how these approaches connect, merge, collapse, and adapt within the evolving context of change and transition [54]. As such, locating participation within processes of change toward transformative spaces of participation requires some reflexivity, making the connection often missing between technological development, structures of governance and the local community worldview [29]. Traditional approaches to engagement to build citizen deliberation into project development have been deemed incoherent in nature and lacking mechanisms to incorporate citizen perspectives [55]. This was echoed in our reflections.

*the approach over the years … was almost just keep the head down and proceed and hopefully that things will just go under the radar as opposed to meeting problems and difficult situations head on and trying to find an engagement pathway through it.* (4)

The above timeline (Fig. 3) depicting key milestones in EirGrid's Journey was developed through the early literature review and reflected upon by some of the participants. A key turning point within this timeline is represented through the publication of the working paper on grid strategy in 2015 ended the pursuit of new developments, instead prioritising the utilisation of existing infrastructure and the redesign of public engagement. At this stage the internal coalition to support the redevelopment of public engagement emerged. In 2017, the updated Grid development strategy was launched, creating a vision for change, and within this the redevelopment of engagement processes significantly impacted upon how projects were brought forward to communities and the relationship between this process and the technical solutions. An increased importance was placed upon such consultation processes with them now holding a greater weight of influence over a project. The communication of the vision towards a new approach to engagement is supported through the development of a new strategy.

*EirGrid has totally changed its approach from one of dictatorial engineer led design to one of meaningful consultation and listening to communities.* (2)

*The primacy of the engineers was traditionally huge in EirGrid, and the optimal technical solution was always the preferred option. Under the new Public Engagement policy, this approach was now being turned on its head and the idea of ‘social acceptance’ was being brought into the process much earlier and carrying significant weight in the process. I was concerned that this might not be accepted but it was not only accepted but appears to have been embraced.* (1)

While the process development since 2015 had been viewed as positive in the whole it is important to recognise the associated enabling factors and challenges still faced, both internally to the organisation and externally at the level of policy, the citizenry, and wider landscape developments.

### 4.2 Levers and obstacles for implementing public engagement strategies

The internal factors which enabled the organisation to shift towards new strategies of engagement came from several different factors (Table 2). The challenging crisis point, whereby new projects could not be developed, acts as a key mechanism through which change could occur. An awareness that something needed to change for the TSO to function moving forward provided a foundational impetus. The creation of the public engagement team and the strong leadership within this was important for building a clear understanding of community needs and the social and political factors associated with them. Central to this journey has been the strong senior governance support from the CEO and CIO (Chief Infrastructure Office) alongside the chairperson and the board. This provided the necessary enabling force to empower action. The technical context is important, with a core aspect of the early success of the strategy related to the increased feasibility of undergrounding of cables for some routes. The creation of such quick wins can embed the new organisational culture of engagement and inform more technically challenging projects, such as the Celtic Interconnector. This undersea cable includes significant onshore construction in relation to the development of a substation. It has followed the established public engagement process and has successfully gained planning without community opposition.

**Table 2:** Internal factors supporting organisational change towards public engagement.

*The thought leadership provided by the company and the prominence of our CE and Senior Executive team in Energy/Climate Action debates further underpinned our credibility*. (2)
*when you're at a crisis, you know, look for that opportunity. (6)*
*Acceptance that there was an important issue in relation to EirGrid's historic approach to Public Engagement that would seriously inhibit EirGrid's delivery of its Strategy, 2020–2025 and the Government's Climate Action Plan unless it was addressed. (1)*
*Agreement to the new policy by the Board and the promulgation of that policy, followed by proper resourcing of the Public Engagement. (1)*
*good public engagement team leadership. A deep understanding of how political, social and community network's function is essential. (2)*
*Working through a learning experience, open communication, and a programmatic approach*. *(3)*
*… undergrounding, this totally changed the narrative. (2)*
*There is no way we would have for this through, in terms of what we're doing now, if at the time we hadn't had the support of a very supportive chairperson*. *(The CEO) has completely bought into it, so that level of senior level support and you will also see it with (the CIO). (6)*

There are also a few internal challenges which the implementation of the public engagement strategy must face (Table 3). The number of projects to be delivered means things are moving fast. This fast pace of project development within the organisation can sometimes be in tension with the time requirements needed to follow the necessary engagement protocols. A central tension within just transitions and energy justice concerns the interplay between rapid low-carbon transitions and participatory processes [56]. In practical terms, it means there is a growing need to create governing structures that can deal with rapid change and anticipate and effectively respond to uncertainty constructively, considering the perspectives of local communities and groups [29]. The cost associated with using underground cabling is also a consideration and on from this there remains in places a conflict between the new approach and more traditional engineering approaches. Finally, the gains that have been achieved in the last number of years, create difficulty in fostering new growth for the future. How can engagement be further developed and improved considering the scale of projects being developed (340+ projects at €3.2Bn)? This creates a challenge in relation to the final phase of Kotter's model, which is making the change stick.

**Table 3:** Internal barriers for implementing public engagement.

*Solutions which evolved which became expectations for future projects. For example, the final solution for the* 40 km *of 400kv AC Kildare Meath project, which was recently lodged for statutory planning, was to underground the line, routed mostly along public roads. However, the technical limit of this technology is* 50 km *and other communities will not accept why they can't have the same solution.* (2)
*Costs are another serious casualty of the process – the outturn cost of an underground solution are likely to be twice that of an overground solution … also the level of resourcing, administratively, people wise and expertise wise required to build out sustainably*. *(2)*
*340 projects in our books to deliver at the moment … the biggest obstacle, it's probably internally because everything is moving so fast that it’s almost impossible to try and catch everything that's happening now*. *(4)*
(There is a) *cultural difficulty at engineering level for a lot of this stuff*. *(5)*
*The fastest growth is always the first stage; when individuals experience is new and serves to build the groups knowledge through sharing. Once a certain level has then been reached, true new growth is required. This is a different type of learning that requires a different skillset and team spirit*. *(3)*

Beyond the internal enabling factors and challenges, there are wider external topics which are important contributors to the success potential of the implementation of the public engagement strategy (Table 4). The wider European context of energy security was referenced as a potential help. The national political context, in which uncertainty existed concerning EirGrid's traditional approach to public engagement, put a necessary pressure on the organisation to undergo this development. The building of collaborative partnerships related to public engagement has also helped facilitate the success of the public engagement approach. One such example is a partnership with Friends of the Earth and Renewable Grid Initiative on the ‘Our Energy Future’ project, seeking to engage communities and groups on Ireland's energy transition. Alongside this, a further collaborative approach related to the ‘Powering Up Dublin’ project brough together all utilities and state bodies associated with the project to establish the Dublin Infrastructure Forum. Here, in relation to implementing and sustaining change, the creation of new partnerships can assist in building on the change developed to date. This outward focus on building a unified approach across agencies can support more holistic engagement practices in Ireland's energy transition.

**Table 4:** External factors enabling public engagement strategy development.

*the thing that has helped in the last while is energy security … I only had this conversation with quite a senior advisor in the government the last day about one of our more problematic projects and it was said to me [saying] “has (energy security) not helped a lot?” And it probably has*. *(5)*
*And I suppose all we can do is use whatever tools are in our toolbox, including the fact that the world has just changed and become more energy conscious and more climate conscious and all that stuff helps. (7)*
*the Government and many elected representatives had concerns about EirGrid's historic approach to Public Engagement. (1)*
*we have some very unusual partnerships that would not have been considered by their organisation years ago*. *(4)*
*So, for the first time ever on a large infrastructure project we brought all of the utilities together, all of the state agencies in Dublin and set up the Dublin Infrastructure Forum and that has been hugely beneficial and it's a model that others are now using and will continue to use it. (3)*

A range of external factors which raise challenges for the implementation of public engagement were referenced through the reflections (Table 5). The scale of new projects which must be developed acts as an internal pressure which is informed by external needs to meet national demands. This is sometimes at odds with the time requirements associated with implementing EirGrid's six-step strategy and the oscillations which may need to occur at different timelines throughout a project. Working with other state agencies in this space can also be a challenge. The Dublin Infrastructure Forum may be an example of an approach through which such issues can be managed. The scale of external opposition to EirGrid in the past was also outlined as a potential challenge moving forward, whereby those previously impacted by poor engagement practices within EirGrid may not trust in the validity of a new strategy.

**Table 5:** External barriers to implementing public engagement strategy.

*There appeared to have been some challenges in getting (other state agencies) to adopt similar types of new processes and to move at the speed that EirGrid thought was required. EirGrid required (other state agencies) to be able to move at the same* pace *or many of the projects would be delayed*. *(1)*
*There was huge disappointment at the assessment by the Commission for the Regulation of Utilities (CRU) of EirGrid's Public Engagement and there was a feeling that CRU did not appreciate the scale and scope of what had been achieved by EirGrid. (1)*
*(We are) under pressure to deliver on (national) 2030 … they are just targets as you know, and the question is well if we were two to three years late but actually had the public bought into this and communities*. *(6)*
*Barriers arose in the form of the Covid pandemic where public contact was curtailed, killing face to face meetings*. *(2)*
*Easy sound bites from a media perspective … It's far, far, far too simplistic the media can do a lot of harm*. *(*7)
*An unexpected barrier arose in the form of Transport Infrastructure Ireland and Local Authority roads engineers. The nature of underground route selection is that public road routing becomes very attractive – it removes multiple landowner conversations, ensures future security, and lessens damage risk exposure. However, road authorities became scheme objectors due to multiple concerns – damage to roads, future costs in road improvements, damage to bridges, etc. (2)*
*It's a new approach within the industry within the sector and indeed within state bodies and what has been externally difficult. Probably just the magnitude of it, quite frankly, because there's so much needs to be done in what is now a very short space of time*. *(4)*
*Legacy projects that had opposition in the past, my fear is that when we move them forward and we commence engagement and we continue to engage on them, that we lose some of that credibility with the public. Their views haven't changed from 10 years ago and I would be fearful that maybe some of the good work that we've done will be undone when we start seeing protests around the country again on some of the legacy projects. And it's difficult for people to believe that we have changed as an organisation. (4)*

## 5 Discussion

### 5.1 Enabling unified approaches to energy transitions through public engagement

Energy transitions do not take place in a vacuum and as such are influenced by a wide array of external factors across numerous sectors of society. These landscape considerations, borrowing from the multi-level perspective, are important considerations when assessing the success potential of an energy transition. Globally, the Covid-19 pandemic and the Russia-Ukraine war have impacted upon the global energy system. Considering such challenges, often short-term responses are implemented, which can impact upon transition and decarbonisation pathways [57]. This reinforces current understandings of public engagement in all its diverse manifestations as emergent processes, which call for more anticipatory and reflexive ways of working collectively and delivering energy system change [29]. When planning for transition timelines out to 2050 there will be considerable landscape challenges to contend with. Furthermore, placing the public at the centre of climate action challenges government-led assumptions of what climate action should be like. Local communities are often expected to fully support broader national climate action goals, such as reducing greenhouse gas emissions, transitioning to clean energy, and changing behaviours toward energy efficiency. Yet, in the context of multiple crises, energy issues may be combined, conflated, or weakened by other priorities [58]. EirGrid's recent effort to work in partnership across institutional boundaries somewhat reflects the need for a more systemic understanding of public engagement with climate action. Working through partnerships with other state organisations and energy agencies in Ireland has provided the opportunity for a more holistic approach.

An example which both contributed to blocking the development of new grid infrastructure in Ireland and enabling the implementation of EirGrid's new public engagement strategy, was the global economic crisis and the Irish government's response to this through the implementation of austerity measures. In this context, the work of EirGrid was associated with the state and not in alignment with a common narrative concerning the national priority of decarbonisation.

A recent report in Ireland found that 81 % of people are either worried or very worried about the climate [59]. However, aclimate exceptionalism approach, which is often blind to other basic human needs such as housing or a living wage, can often disenfranchise people towards action. Due to this, there is a need for a clear narrative concerning Ireland's transition pathway; what are the benefits and trade-offs? How will the energy system change? What is the role of different actors? Within the reflections outlined through this work, it was acknowledged clearly that people on the ground are often primarily concerned with local issues in relation to the development of infrastructure. EirGrid, however, has played a role in contributing to the socialisation of a national narrative concerning the energy transition. This is not to suggest it as a success, but rather that agencies within the energy sector may have a key role to play in convening relevant parties to clearly outline the national energy priorities. Through public engagement processes such as the Energy Citizen Roadshows and the Shaping Our Electricity Future process (Boyle et al., [60]), EirGrid have taken on a role in this, far removed from their primary or traditional function in developing and operating the electricity transmission system. The failure to create a unified narrative - which can be co-created to some degree with the citizenry - concerning the direction of travel related to the energy system will leave the success potential of the transition open to landscape impacts beyond control.

### 5.2 Slowing down to speed things up

The need to respond urgently to the global ecological crisis is coupled with the creation of national targets which are framed in relation to specific dates i.e., 2030, 2050 etc. While the rationalist logic of this approach is clear, it may warrant some critical reflection due to the widespread inaction currently experienced [[61], [62]]. While European greenhouse gas emissions are expected to drop by 2 % from 2021 levels, cumulatively, global emissions continue to rise, and the broader ecological context of the crisis remains. In reflecting upon the engagement journey to date, the conflict between this policy necessity towards targeted reductions and the implementation of genuine public engagement strategy is a tension in need of greater consideration. Failure to meet decided target timelines by a year or two due to the implementation of the necessary engagement strategy to get grid infrastructure built is a more advantageous position to be in compared with undertaking cursory engagement and consultation processes which lead to widespread opposition to a project which can set a project back by decades. This again can relate in some sense to the creation of a unified approach to energy transitions facilitated through following the necessary public engagement approach. This is a critical crossroads in delivering energy system change at an accelerated rate and in many ways, it represents an effort to ‘sustaining democratic governance in hard times’ ([63], 251). Faced with opposition and the urgent need to deliver energy system change we see a tension between calls for soft environmental democracy processes or harder eco-authoritarianism style delivery. EirGrid's challenge aligned to democratic innovations in this context is to balance this process with other technical and organisational requirements. All of which are both prone and vulnerable to delays and time constraints [63].

Within the project management team, this acceptance that public engagement may be an iterative process which may oscillate through its implementation can prove difficult to garner. The traditional linear logic of project development and delivery within technically dominant infrastructure developers is inattentive to the non-linear and uncertain nature of societal responses to infrastructure developments if the necessary procedures are not followed in relation to public engagement. The senior management acceptance of the need to get projects built and the societal dimensions of this, irrespective of the technically optimum solution, is a key reason for the successful implementation of EirGrid's public engagement strategy to date. This, however, remains a tension. A lack of understanding of the societal dimensions of infrastructure development remains an important concern [64]. The need to coherently explain the time required for public engagement, and the conflict this can potentially have with linear project delivery approaches, is crucial to the successful implementation of an effective public engagement strategy.

### 5.3 Early adopters, engagement, and navigating pitfalls

One of the respondents within the reflective process acknowledged the well-worn wisdom within organisational studies that organisation change is hard and requires the necessary antecedent conditions, the engagement of key stakeholders within the organisation, an understanding of internal and external factors driving change, and adequate resources and capacity building. The respondent traced this back to *The Prince*;

“There is nothing more difficult to take in hand, more perilous to conduct, or more uncertain in its success, than to take the lead in the introduction of a new order of things. For the innovator has enemies in all those who profit by the old order, and only lukewarm defenders in all those who would profit by the new order, this lukewarmness arising partly from fear of their adversaries … and partly from the incredulity of mankind, who do not truly believe in anything new until they have had actual experience of it.” (Machiavelli, quoted in [65]).

The concept of early adopters is well developed within the transition management literature in relation to the diffusion of technological innovation and behaviour change [66]. Further work is needed on early adopters of legitimate public engagement strategies in relation to the development of energy infrastructure. As we begin to learn more about the dynamics of implementing such strategies as early adopters the potential to facilitate a large early majority increases.

EirGrid's challenges between 2013 and 2015 in relation to public opposition to their processes resulted in the organisation not being able to act according to its modus operandi. As early adopters, some of the lessons contained within this reflective exercise may enable other entities to undergo similar strategic, structural, and personnel changes in relation to public engagement without undergoing the same reputational scrutiny and operational strain. A vast array of entities related to energy infrastructure (TSOs, DSOs, wind, hydrogen, gas etc.) may face societal challenges when seeking to build infrastructure for the energy transitions. While nothing is to ensure success, a well-developed public engagement strategy may support and inform project development and contribute to wider societal engagement in relation to energy transitions.

## 6 Conclusions

In previously discussing engagement processes within state agencies and public bodies concerned with the delivery of infrastructure aligning with climate action targets and policies, it was noted that a structured approach to engagement and public participation which is transparent, inclusive, and democratic is needed to mitigate institutional distrust and widespread opposition to strategic infrastructure [4]. Attempts at such engagement practices must be willing to embed societal input into project governance, balancing it with technical and economic considerations. This paper has highlighted the institutional journey of a European TSO in relation to public engagement, reorientating the organisational culture towards early and continued engagement with citizens and communities.

To comprehensively assess EirGrid's progress towards more inclusive and deliberate decision-making processes, we have formulated a set of guiding questions with a focus on achieving meaningful resolutions from a practitioner's lens. A key concluding question therefore is- how can this reflective process inform and influence future actions, rather than simply serving as a historical record of past decisions and events? We have seen that a key enabler in EirGrid's journey to new and more experimental public engagement strategy relates to both political and administrative readiness to embrace new partnerships and ways of delivering change. Ensuring the skills and legacy of this journey can be converted into future learning is critical and as such the reflective practice process itself has provided the means to facilitate future action. The evidence also shows that top management has been an influential mediator in the establishment of these new relationships and their role is likely to continue to hold a strong influence given the internal tensions between aligning the more technical aspects of project delivery and the public engagement process. Looking ahead, we can also gauge from the reflections that the large volume of new projects and the reworking of problematic legacy projects present numerous challenges matched by further constraints to align this work with other institutional priorities (such as the road authority). The growing role of partnership and exchange through media channels should be central in further public strategies working together to ensure co-benefits are realised and are properly communicated with all stakeholders.

The significance of integrating communities and individuals in the energy transition is becoming increasingly apparent in national and European policy. Some lessons have emerged from this reflective practice process to inform this stated need. Emergent engagement practices can conflict with incumbent technically focused approaches within an organisation, and as such, senior leadership is essential. The business-as-usual model runs the risk of bringing forward oppositional campaigns. Organisational change towards new practices of public engagement is needed.

While the new practices outlined in this paper emerged through responding to crisis, other organisations tasked with the delivery of key infrastructure to meet 2030 targets will not have the benefit of crisis and, as such, must proactively seek to reform their internal public engagement practices. Finally, the depth of engagement and time required to support these processes is not aligned with the urgent pace of infrastructural changes needed to meet European emissions reduction targets. Attempts to streamline engagement to the timelines of targets may be perilous. The relationship between public engagement and energy transitions draws parallels to the tortoise and the hare [67] and researchers and practitioners must reflect more deeply on the scale of transformation required and the level of citizen participation needed.

## CRediT authorship contribution statement

**Evan Boyle:** Writing – original draft. **Alexandra Revez:** Writing – review & editing. **Aoife Deane:** Writing – review & editing. **Brian Ó Gallachóir:** Writing – review & editing.

## Declaration of competing interest

The authors declare that they have no known competing financial interests or personal relationships that could have appeared to influence the work reported in this paper.
