# Supplementary material for: Levers and obstacles for implementing public engagement practices in electricity grid development
Source: Heliyon. 2024 Jul 23;10(15):e34955. doi: 10.1016/j.heliyon.2024.e34955 (PMC11336281; doi:10.1016/j.heliyon.2024.e34955)
Supplement: Multimedia component 1 [file mmc1.docx]

Dear Prof. Mo Ng,

The research team has now added are responses to the comprehensive review comments which we have received. We really appreciated the reviewers time to help strengthen this paper and their positive feedback in relation to its value. We have added depth in relation to the theoretical dimensions associated with public engagement and organisational change. The dimensions of organisation change have been brought through to the results and theoretical aspects of public engagement are developed further in the discussion and conclusion. Within this, we have sought to more clearly focalise the importance and insights garnered from this research for other organisations due to undertake similar processes and the relevance of such to future sustainable development in the context of delivering infrastructure associated with the energy transition and climate action measures more broadly. The table below highlights responses and additions throughout. Additions made are referenced in red.

Thank you,
Evan

| R1. This is a really interesting piece which has a lot of learning for those who are actively engaged in changing organisational cultures and practices around public engagement in large organisations responsible for infrastructural projects that are likely to generate public opposition. The reflective element is done well but more theory needs to be embedded in the piece to bring out all the learnings from it and the added value of it. | Thank you |
| --- | --- |
| R2. This is an interesting and important topic. The authors outline the need for transmission systems upgrading to facilitate clean energy electrification targets, and replace aging infrastructure. They outline the history and context of community opposition to the Irish TSO's upgrading plans, and the TSO's revision of their public engagement strategy after criticism of tokenism. The paper reflects on the Irish TSO's organisational journey towards developing revised public engagement strategies, procedures, and processes. The context is outlined well. The document is well structured, the English language usage throughout the document needs to be polished, samples below. | Thank you |
| REVIEWER ONE |  |
| It will be essential of your work is delivering a clear direction of future sustainable development. Please consider to emphasize by developing in your discussion section | Thank you, we have sought to make additions through Section 5 to enhance to application of the findings in relation to future sustainable development.   - This is a critical crossroads in delivering energy system change at an accelerated rate and in many ways, it represents an effort to ‘sustaining democratic governance in hard times’ (Fischer, 2017, p.251). Faced with opposition and the urgent need to deliver energy system change we see a tension between calls for soft environmental democracy processes or harder eco-authoritarianism style delivery. EirGrid’s challenge aligned to democratic innovations in this context is to balance this process with other technical and organisational requirements. All of which are both prone and vulnerable to delays and time constraints (ibid). - This reinforces current understandings of public engagement in all its diverse manifestations as emergent processes, which call for more anticipatory and reflexive ways of working collectively and delivering energy system change (Revez et al. 2022). When planning for transition timelines out to 2050 there will be considerable landscape challenges to contend with. Furthermore, placing the public further at the centre of climate action challenges government-led assumptions of what climate action should be like. Local communities are often expected to fully support broader national climate action goals, such as reducing greenhouse gas emissions, transitioning to clean energy, and changing behaviours toward energy sobriety. Yet, in the context of multiple crises, energy issues may be combined, conflated, or weakened by other priorities (Mullally et al. 2022). EirGrid’s recent effort to work in partnership across institutional boundaries somewhat reflects the need for a more systemic understanding of public engagement with climate action. Working through partnerships with other state organisations and energy agencies in Ireland has provided the opportunity for a more holistic approach. |
|  |  |
| Please ensure that your Highlights, Title and Abstract are amended to reflect any major changes you made to your paper as a result of the reviewers’ comments and suggestions. Highlights consist of a short collection of bullet points that convey the core findings of the article and should be submitted in a separate file in the online submission system. Please use 'Highlights' in the file name and include 3 to 5 bullet points (maximum 85 characters, including spaces, per bullet point). See the following website for more information <http://www.elsevier.com/highlights> | Changes made to abstract and title. The use of highlights has been added to provide a succinct insight into the value of the paper for potential readers.  Highlights  - Documents the barriers and drivers for the development of public engagement strategies for organisations tasked with infrastructure delivery.  - An internal reflective practice process across a transmission system operator to provide an analysis of organisational change in relation to public engagement.  - Highlights the tension between speeding up the low-carbon transition and engaging citizens in transition processes.  - Public engagement must be strongly embedded within organisations in order to influence the technical aspects of project delivery. |
| If applicable, please ensure the dates and location of your study are stated in your methods section.  Discussion/conclusion: a major factor in consideration of accepting your paper is to delivering a clear message in future direction of sustainability in the subject area. Practical implications provided in future research, policy and relevant industry is essential in your discussion section. | Year 2022-2023 added.  Some alterations have been made to both the discussion and the conclusion as referenced again below.  1.. This reinforces current understandings of public engagement in all its diverse manifestations as emergent processes, which call for more anticipatory and reflexive ways of working collectively and delivering energy system change (Revez et al. 2022). When planning forOver transition timelines out to 2050 there will be considerable landscape challenges to contend with. Furthermore, placing the public further at the centre of climate action challenges government-led assumptions of what climate action should be like. Local communities are often expected to fully support broader national climate action goals, such as reducing greenhouse gas emissions, transitioning to clean energy, and changing behaviours toward energy sobriety. Yet, in the context of multiple crises, energy issues may be combined, conflated, or weakened by other priorities (Mullally et al. 2022). EirGrid’s recent effort to work in partnership across institutional boundaries somewhat reflects the need for a more systemic understanding of public engagement with climate action. Working through partnerships with other state organisations and energy agencies in Ireland has provided the opportunity for a more holistic approach.  2… This is a critical crossroads in delivering energy system change at an accelerated rate and in many ways, it represents an effort to ‘sustaining democratic governance in hard times’ (Fischer, 2017, p.251). Faced with opposition and the urgent need to deliver energy system change we see a tension between calls for soft environmental democracy processes or harder eco-authoritarianism style delivery. EirGrid’s challenge aligned to democratic innovations in this context is to balance this process with other technical and organisational requirements. All of which are both prone and vulnerable to delays and time constraints (ibid).  In conclusion:  To comprehensively assess EirGrid’s progress towards more inclusive and deliberate decision-making processes, we have formulated a set of guiding questions with a focus on achieving meaningful resolutions from a practitioner’s lens. A key concluding question therefore is -How can this reflective process inform and influence future actions, rather than simply serving as a historical record of past decisions and events? We have seen that a key enabler in EirGrid’s journey to new and more experimental public engagement strategy relates to both political and administrative readiness to embrace new partnerships and ways of delivering change. Ensuring the skills and legacy of this journey can be converted into future learning is critical and as such the reflective practice process itself has provided the means to facilitate future action. The evidence also shows that top management has been an influential mediator in the establishment of these new relationships and their role in likely to continue to hold a strong influence given the internal tensions between aligning the more technical aspects of project delivery and the public engagement process. Looking ahead, we can also gauge from the reflections that the large volume of new projects and the reworking of problematic legacy projects present numerous challenges matched by further constraints to align this work with other institutional priorities (such as the road authority). The growing role of partnership and exchange through media channels should be central in further public strategies working together and ensure co-benefits are realised and are properly communicated with all stakeholders. |
| Can a few of the key learnings be mentioned and the resulting implications for practice. | See above, key highlights also added.. |
| Introduction  Can statements like the below be referenced using either the literature or the grey literature?  1. While best practice is often called for, the context specific nature of engaging with diverse publics, gives the need for a more measured approach whereby "good practice" can inform the ways in which citizens and other stakeholders are engaged with.  2. Alongside this, however, is the need for flexibility, in responding to different contexts, circumstances, and changes throughout a project timeline.  3. The call for greater participation is premised on the considerable and complex political challenges faced at a global scale, such as polarization, disinformation, geo-political tensions, and climate change.  4. Can you reference the definitions of normative and pragmatic participation?  5. The core aim of the paper is only provided in the last sentence of this introduction.  Here, we reflect upon EirGrid, as the national grid operator, and the organisations journey developing new and innovative engagement strategies for grid development in the Irish context.  6. Can you your added value over other studies be explained and why it's important to reflect on cases such as this one.  7. If you have research questions, can these be provided here also; their added value and importance.  8. Detail the structure of the paper for the reader. | 1. Added Boyle et al., 2022 Energy Policy 2. Reference moved for clarity 3. Added Sacks 2020, Hachette UK 4. See: Reed, 2008, Biological conservation 5. Section Moved to first paragraph with new closing sentence of introduction put in place. 6. We have added a section here which outlines specifically the relevance of the paper. “A recent IEA report (2023) on public engagement in energy infrastructure found that no studies to date have investigated the drivers and barriers of institutions engaging with citizens and communities. Beyond this, no research has taken place which highlights the organisational development of new public engagement practices within institutions tasked with developing infrastructure associated with the energy transition. This creates a challenge whereby ever-increasing calls for public engagement are made, but no lessons exist with relation to how such new practices can be embedded within an organisational strategy. In providing reflections from within a TSO we develop knowledge in this space.” 7. The research question has now been explicitly outlined as such “what are the key levers and barriers for organisation change towards new forms of public engagement in infrastructure delivery?” This has been formulated in the introduction, abstract and conclusions. 8. We have added a section which outlines the structure of the paper which is included within the last paragraph of the introduction:   “This paper reflects upon the implementation of new engagement strategies for electricity grid development through an analysis of EirGrid and their emergent practice, answering our central research question: what are the key levers and barriers for organisation change towards new forms of public engagement in infrastructure delivery? The structure of the paper is as follows: firstly, we outline the contextual specifics of EirGrid, as Irelands transmission system operator, in relation to public engagement. We briefly outline theoretical insights in relation to both engagement and organisational change which support the development of this paper. Following this, the methods section outlines the use of a reflective practice process. On from this, we develop results related to the reflective process in relation to the engagement journey so far, levers and obstacles to public engagement, and conclusions. In the discussion section, we focus on the need for unified approaches to public engagement at a cross-organisational level to deliver on the energy transition, the tension between meeting climate targets and engaging with citizens, and implementing new engagement processes at an early stage. Finally, we draw out conclusions and implications for future developments in relation to sustainability and infrastructural delivery”. |
| Context: Developing a strategy for Public Engagement within a Transmission System Operator  1. Again here can you link to grey literature to support points like - Northern Ireland all saw the mobilisation of significant community-based opposition and the networking of different oppositional groups within this.  2. The development of the context is very useful, but you are reflecting on organisational change and there would be an expectation that you would consider theories underlying organisational change. - systems theory, organisational development theory, complexity theory and social worlds theory  3. as well as theories underlying public engagement e.g. A theory of participation: what makes stakeholder and public engagement in environmental management work? (wiley.com) | 1. This is referenced through the article (Mullally & Byrne, 2016). 2. The following is added- “Lewin’s foundational change management model, which outlines change across three distinct phases, involves the disentanglement of previous practice, the change taking place, and embedding new practices through a process of permanentization (Cummings et al., 2016). Beyond this linear pathway, more recent complexity theories adds great depth, whereby processes of change are often emergent with small changes having the potential to lead to larger system level outcomes (Burnes, 2005). These two fields of theoretical analysis related to organisational change offer opposing points on a spectrum representing linear and non-linear change. The work of John Kotter (1996), developed through his 8-step change model, is referenced through the different stages of the process as reflected upon by participants. Within this the importance of unintended outcomes as represented by complexity theories is also noted”- This is carried over to the analysis 3. Added “At a theoretical level, while no single definition of public engagement can suitably encapsulate the range of forms which it takes, it is being more deeply developed in relation to emergent conceptions of citizenship over passive notions of consumer choice due to the increasing relevance of societal dimensions of transition processes and the political and social factors contained within (Revez et al., 2022)”. |
| Methods  1. Can Figure 2 be placed on a landscape page and the font increased. Can it be referred to in the text? Can it be discussed and explained in the context of Gibbs approach.  2. Can the open reflective questions or themes be discussed and how these may bound or otherwise the nature of the reflection. The added value of these needs to justified in the introduction. If the latter needs to be done here an argument should be provided in the response to support same.  3. Can more information be provided on the interviewees (gender/age .. length of time within the organisation) or reasons provided why this cannot be disclosed? Also can any biases be explained which may arise as a result | 1. Landscape orientation. Added reference to (fig.2). Added the following in relation to Gibbs work. “The Gibbs model has been built upon to include specific questions (see fig. 2) in order guide the reflections beyond the broad foundations outlined by Gibbs. Building on Gibbs, the incorporation of specific questions (see fig. 2) is used to support the respondents due to the novel nature of such a reflective endeavour within the organisation”. 2. Agree. We added two sentences to the introduction section outlining the key research question framing our paper within a reflective practice lens.   “In this paper, we are examining the reflections of practitioners paving the way for organizational change and implementing new forms of public engagement in infrastructure delivery. Our primary focus is to identify the key drivers and barriers for organisational change in this context. Moving from policy to implementation is a key development for the integration of deliberative processes into governance. Here, we reflect upon EirGrid, as the national grid operator, and the organisations as the national grid operator and the organisation on its journey to developing new and innovative engagement strategies for grid development in the Irish context”   3. This is a good point and something which we had to balance with regards to working with participants through the ethics proposal/ ethics form. The following few lines are given to clarify are considerations in this regard. “Some biases or conflicts of interest may exist with regards to undertaking such a reflective practice within an organisation with active staff members. To give an open space for critical reflection, the reflections provided are not directly attributed to individuals as this would make them clearly identifiable within the Irish context (e.g. chairperson etc.). This was outlined within the ethics form provided to participants. The findings from practitioner reflections may be limited as they heavily depend on their willingness and ability to evaluate their practices. While the practitioners we have worked with have been open and cooperative during the research process, there may still be limitations or biases in how they assess their performance. Despite these limitations, we believe that the results can be useful in other contexts. Many European countries, including Ireland, have faced challenges in fully embracing more extensive forms of public engagement, and some are just beginning to explore this area. Therefore, there is value in learning from the experiences of EirGrid, as shared by the practitioners involved, as it can provide valuable insights and opportunities for others. ” Alongside this information in a cursory way has been provided on gender: “Experience within the organisation ranged from 2 years to 15+ years. The gender balance represented was 4 male 3 female participants”. |
| Results  1. Can individuals 1-7 be identified vis their role - community liaison officer, chairperson, non-executive director, senior planning officer, head of public engagement, external affairs, and head of network development.  2. Can Figure 3 be placed on a landscape page and the font increased. Can it be referred to in the text? Consider placing it the context section as this would be useful there.  3. The findings in this section are very descriptive at present. Comment on the learnings from the analysis by theme. Link/reflect on these based on theories of engagement, organisational change (and potentially social acceptance).  4. Refer to the tables in the text and the evidence provided in them to support the learnings. | 1. This is related again to point 3 above. 2. I have now moved it to landscape and also made reference to it within the text. I have kept it within the results section as it was a useful tool for reflection and helps the reader see the range of changes as they are being introduced in section 4.1. “The above timeline (fig.3) was developed through the early literature review and reflected upon by some of the participants. A key turning point within this timeline is represented through tThe publication of the working paper on grid strategy in 2015 ended the pursuit of new developments, instead prioritising the utilisation of existing infrastructure and the redesign of public engagement” 3. A number of additions have been made throughout linking reflections to literature and theory in relation to engagement where relevant:  - “This fast pace of project development within the organisation can sometimes be in tensions with the time requirements needed to follow the necessary engagement protocols. A central tension within just transitions and energy justice concerns the interplay between rapid low-carbon transitions and participatory processes (Newell et al., 2022)”. - “Traditional approaches to deliberative engagement have been deemed incoherent in nature and lacking mechanisms to incorporate citizen perspectives (Cotton &Devine-Wright, 2010). This was echoed in our reflections.” - In advocating for a more interconnected and interactive definition of public engagement, recent research has highlighted the necessity for a new dialogue. This dialogue should be less concerned with ranking the merits of specific public engagement approaches and more focused on understanding how these approaches connect, merge, collapse, and adapt within the evolving context of change and transition (Pallett, Chilvers and Hargreaves, 2019). As such, locating participation within processes of change toward transformative spaces of participation requires some reflexivity, making the connection often missing between technological development, structures of governance and the local community worldview (Revez et al., 2022). - In practical terms, it means there is a growing need to create governing structures that can deal with rapid change and anticipate and effectively respond to uncertainty constructively, considering the perspectives of local communities and groups (Revez et al. 2022).   We have also used Kotter (1996) to reference the organisational change outlined through the reflections in relation to his model of change. See the new Table 1 at the beginning of the results. Through 4.1 and 4.2 we make reference to the different stages across this model.   4. References have now been made to tables 2-5 in the corresponding paragraphs. Alongside this we have removed 4.3 on conlcusions and looking forward to give more direct focus to the journey so far and internal levers and obstacles. From this we draw conclusions later. |
| Discussion  1. There should be a link between the learnings under the themes in the results section to the subsections of the discussion where you are proposing elements to consider when undertaking organisational change surrounding approaches to public engagement. Bring in more links to theory here also.  2. Conclusion - You could be more specific about the nature of the reform in engagement practices that this reflection suggests. | 1.. This reinforces current understandings of public engagement in all its diverse manifestations as emergent processes, which call for more anticipatory and reflexive ways of working collectively and delivering energy system change (Revez et al. 2022). When planning forOver transition timelines out to 2050 there will be considerable landscape challenges to contend with. Furthermore, placing the public further at the centre of climate action challenges government-led assumptions of what climate action should be like. Local communities are often expected to fully support broader national climate action goals, such as reducing greenhouse gas emissions, transitioning to clean energy, and changing behaviours toward energy sobriety. Yet, in the context of multiple crises, energy issues may be combined, conflated, or weakened by other priorities (Mullally et al. 2022). EirGrid’s recent effort to work in partnership across institutional boundaries somewhat reflects the need for a more systemic understanding of public engagement with climate action. Working through partnerships with other state organisations and energy agencies in Ireland has provided the opportunity for a more holistic approach.  2… This is a critical crossroads in delivering energy system change at an accelerated rate and in many ways, it represents an effort to ‘sustaining democratic governance in hard times’ (Fischer, 2017, p.251). Faced with opposition and the urgent need to deliver energy system change we see a tension between calls for soft environmental democracy processes or harder eco-authoritarianism style delivery. EirGrid’s challenge aligned to democratic innovations in this context is to balance this process with other technical and organisational requirements. All of which are both prone and vulnerable to delays and time constraints (ibid).  In conclusion:  To comprehensively assess EirGrid’s progress towards more inclusive and deliberate decision-making processes, we have formulated a set of guiding questions with a focus on achieving meaningful resolutions from a practitioner’s lens. A key concluding question therefore is -How can this reflective process inform and influence future actions, rather than simply serving as a historical record of past decisions and events? We have seen that a key enabler in EirGrid’s journey to new and more experimental public engagement strategy relates to both political and administrative readiness to embrace new partnerships and ways of delivering change. Ensuring the skills and legacy of this journey can be converted into future learning is critical and as such the reflective practice process itself has provided the means to facilitate future action. The evidence also shows that top management has been an influential mediator in the establishment of these new relationships and their role in likely to continue to hold a strong influence given the internal tensions between aligning the more technical aspects of project delivery and the public engagement process. Looking ahead, we can also gauge from the reflections that the large volume of new projects and the reworking of problematic legacy projects present numerous challenges matched by further constraints to align this work with other institutional priorities (such as the road authority). The growing role of partnership and exchange through media channels should be central in further public strategies working together and ensure co-benefits are realised and are properly communicated with all stakeholders. |
| Minor  There are many typos throughout e.g. Plans are in place across Europe to decarbonize energy systems with increased importance placed on the generation of renewable electricity. Line 1, para1, pg 1 or line 3 para1, pg 1 12 % should read as 12% or Line 4, para1, pg 1, Despite these developments-; remove the '-'  Can the paper be thoroughly proofread.  Watch your numbering of sections.  A statement on ethical access to the data would help also. | We have thoroughly proof read paper and updated numbers of sections. |
| REVIEWER TWO |  |
| Overall, there are some major weaknesses: 1) The authors need to make a better link to their Method and Results by first explicitly stating the paper's objective, the research gap and the research question. The abstract promises to develop "key findings related to the conflict between engagement practices and traditional project delivery, the need for cross-institutional collaborations towards citizen participation in energy transitions, and the tension between deep engagement and an expedited energy transition."….but there was no analysis of the conflict between practices, and very little was said about cross-institutional collaboration. The Results only addressed the revised engagement strategy. | 1. Thank you for the comment and it aligns with the feedback from another reviewer. As such we have sought to more clearly outline the research question and the research gap.   “what are the key levers and barriers for organisation change towards new forms of public engagement in infrastructure delivery?”  and  “A recent IEA report (2023) on public engagement in energy infrastructure found that no studies to date have investigated the drivers and barriers of institutions engaging with citizens and communities. Beyond this, no research has taken place which highlights the organisational development of new public engagement practices within institutions tasked with developing infrastructure associated with the energy transition. This creates a challenge whereby ever-increasing calls for public engagement are made, but no lessons exist with relation to how such new practices can be embedded within an organisational strategy. In providing reflections from within a TSO we develop knowledge in this space”  Centrally important here and in response to your comment, we have reorientated our abstract to be more specific to what this paper achieves. We provide insights on an organisational shift towards new and more strategically embedded public engagement approaches. Findings from this have relevant to other TSOs or Institutions and as such this is outlined specifically. |
| 2) The Method used is self-reflection - but the reader does not know the questions the participants were asked to reflect on - hence the responses are difficult to put into context. | 2. Thank you this is a good point to raise. We sought to make this clearer to the reader directing towards fig. 2 which outlines specific questions. We have also enhanced the size of fig. 2 and fitted to landscape in page orientation.  “Here, we have designed a six- step reflective processes (fig.2) informed by Gibbs which takes place over a two-month timeframe with each respondent journaling thoughts over each category. The Gibbs model has been built upon to include specific questions (see fig. 2) in order guide the reflections beyond the broad foundations outlined by the model. Building on this, the incorporation of specific questions (fig. 2) is used to support the respondents due to the novel nature of such a reflective endeavour within the organisation.” |
| 3) The reader does not have any insight on how the comments are selected from all the responses - they seem to be selective - we get no sense of where there was a diversity of opinion - something we would expect to see in organisation transformation. | Thank you, we had not provided the necessary information here. Changes have now been made to the methods section to give a better understanding of our approach to data analysis:  “The constant comparative method of analysis (Boeije, 2002) was deployed across the seven reflections developed by participants. Selective coding was used to understand the themes with most pertinence to the development of public engagement strategy within the organisation. This created two main groupings: internal levers and barriers and external levers and barriers. Alongside this more general reflections, aligning with a chronological depiction of the process within the organisation was developed (see: fig.3).” |
| 4) The context and political and economic situation in Ireland are interesting - what are the insights for an international audience from the study who may also have engineering focused TSOs and institutions? | We have made changes to both the discussion and conclusion which aligns with the application of lessons to other contexts. The following section has also been added to the contextual section at the beginning of the paper.  “EirGrid’s journey is deeply connected to the Irish socio-political, economic, and even geographical position as an Island country in the periphery of Europe. Yet, the story of overreliance on technical and engineering solutions to deliver energy grid upgrades and public protest emerging from overreliance on such methods is not a specific Irish issue and we see this problem replicated across various other countries in Europe and the US (Cotton and Devine-Wright, 2012; Komendantova and Battaglini, 2016; Kunreuther et al., 1996).  Decide-Announce-Defend models or eco-authoritarianism style delivery of grid projects have led to lack of agreements over deployment and to costly delays in the development and cancellation of projects (Komendantova and Battaglini, 2016). Contrary to more reductive views of communities as too focused on local issues and incapable of understanding the ‘big picture’ (Ballo 2015) we see from this study how it is possible to include some innovations that provide a more integrated approach to manage the delivery of grid expansion from a multiple scale and stakeholder perspective. The emphasis of these reflections as coming from an Irish practitioners journey to deliver timely energy system change can be interpreted as an invitation for others to share, reflect or critique this geographically bound study and its applicability elsewhere.” |
| 5) The authors mention "strong leadership" as a factor but then say " a core aspect of the success of the strategy to date related to the increased feasibility of undergrounding of cables for some routes"…..it became technically feasible to do what the community asked for. It would seem the new engagement was not the reason at all. | This is a very good point and we were not clear on the importance of this in relation to quick wins. We have now used Kotter’s model of organisational change to indicate this technical feasibility as a contributing factor for highlighting public engagement process using quick wins.  “The technical context is important, with a core aspect of the early success of the strategy to date related to the increased feasibility of undergrounding of cables for some routes. The creation of such quick wins can embed the new organisational culture of engagement and inform more technically challenging projects, such as the Celtic Interconnector. This undersea cable includes significant onshore developments with relation to the development of a substation. It has followed the established public engagement process and has successfully gained planning with little community opposition”. |
| 6) Noting that such institutions have historically been male dominated - is there a gender dimension to this institutional transformation from technical engineering problem solving to embracing "social acceptance"? | This is a limitation of the paper and was not a point under consideration. Paricipants in the reflective process were a 4/3 male-female split. This has now been referenced. |
| 7) The Discussion and Conclusions are not well linked to the idea of organisation transformation. | The following changes have been made to the discussion and conclusion. The findings have also been now linked more closely to theories of organisational change (i.e. Kotter)  This reinforces current understandings of public engagement in all its diverse manifestations as emergent processes, which call for more anticipatory and reflexive ways of working collectively and delivering energy system change (Revez et al. 2022). When planning forOver transition timelines out to 2050 there will be considerable landscape challenges to contend with. Furthermore, placing the public further at the centre of climate action challenges government-led assumptions of what climate action should be like. Local communities are often expected to fully support broader national climate action goals, such as reducing greenhouse gas emissions, transitioning to clean energy, and changing behaviours toward energy sobriety. Yet, in the context of multiple crises, energy issues may be combined, conflated, or weakened by other priorities (Mullally et al. 2022). EirGrid’s recent effort to work in partnership across institutional boundaries somewhat reflects the need for a more systemic understanding of public engagement with climate action. Working through partnerships with other state organisations and energy agencies in Ireland has provided the opportunity for a more holistic approach.  2… This is a critical crossroads in delivering energy system change at an accelerated rate and in many ways, it represents an effort to ‘sustaining democratic governance in hard times’ (Fischer, 2017, p.251). Faced with opposition and the urgent need to deliver energy system change we see a tension between calls for soft environmental democracy processes or harder eco-authoritarianism style delivery. EirGrid’s challenge aligned to democratic innovations in this context is to balance this process with other technical and organisational requirements. All of which are both prone and vulnerable to delays and time constraints (ibid).  In conclusion:  To comprehensively assess EirGrid’s progress towards more inclusive and deliberate decision-making processes, we have formulated a set of guiding questions with a focus on achieving meaningful resolutions from a practitioner’s lens. A key concluding question therefore is -How can this reflective process inform and influence future actions, rather than simply serving as a historical record of past decisions and events? We have seen that a key enabler in EirGrid’s journey to new and more experimental public engagement strategy relates to both political and administrative readiness to embrace new partnerships and ways of delivering change. Ensuring the skills and legacy of this journey can be converted into future learning is critical and as such the reflective practice process itself has provided the means to facilitate future action. The evidence also shows that top management has been an influential mediator in the establishment of these new relationships and their role in likely to continue to hold a strong influence given the internal tensions between aligning the more technical aspects of project delivery and the public engagement process. Looking ahead, we can also gauge from the reflections that the large volume of new projects and the reworking of problematic legacy projects present numerous challenges matched by further constraints to align this work with other institutional priorities (such as the road authority). The growing role of partnership and exchange through media channels should be central in further public strategies working together and ensure co-benefits are realised and are properly communicated with all stakeholders. |
| Minor points and typos Introduction: importance place -> importance placed…, electrification grids->electrification, grids are…, a recognition with the_ a recognition within the ….. Check the full document Clarify "12 % increase in its transmission grid"….increase in capacity or Km of cables or other measure? Section 2. in Irish….in Ireland? | Full document has been proof-read with changes marked. |
| Method: experiential-intuitive model reflective practice with the TSO and author's research group aiming to achieve reflection-for-action asking " How can this process of reflection inform moving forward rather than just document looking backwards? ". We're told that the participants span a cross section of the company - but not how they were selected/or facilitated (by the TSO) to participate in the review. Are the authors certain the participants are the correct set to capture the relevant perspectives? What are the selected responses in Table 2 a reflection on - what were the participants asked? | The following outline has been provided to give more clarity concerning how the reflective cycle is used to shape findings aas they are represented.  “The reflections provided in 4.1 were outlined in the “description” and “feeling” phases of the reflective cycle. For section 4.2 the “evaluation” and “analysis” phases. The “conclusion” and “action plan” are not directly represented in results, but informed the two sections below and are used in shaping the discussion in section 5”.  We have made improvements to the figure on reflection cycle which more clearly highlights the questions asked of participants. |
| Fig.s 2 and 3: The font is too small to read. Section 3 Results is labelled as Section 2 with subheadings 4.X. Justify why these three themes were chosen to synthesis the reflections. Explain who "Friends of the Earth" and other organisations are - add references so the reader understands the objectives of these groups. Section 4 Discussion is labelled as Section 3 with subsections 5.x References some items are missing, e.g. IEA, 2023. Ensure all references are included. | We have made changes here to make it more readable. |
